# Metformin Ameliorates Inflammation and Airway Remodeling of Experimental Allergic Asthma in Mice by Restoring AMPK*α* Activity

**DOI:** 10.3389/fphar.2022.780148

**Published:** 2022-01-24

**Authors:** Wenxian Ma, Qiaoyan Jin, Haiqin Guo, Xinpeng Han, Lingbin Xu, Shemin Lu, Changgui Wu

**Affiliations:** ^1^ Department of Pulmonary and Critical Care Medicine, Xijing Hospital, Fourth Military Medical University, Xi’an, China; ^2^ Department of Biochemistry and Molecular Biology, School of Basic Medical Sciences, Xi’an Jiaotong University Health Science Center, Xi’an, China; ^3^ Department of Pediatrics, The Second Affiliated Hospital of Xi’an Jiaotong University, Xi’an, China; ^4^ Department of Pulmonary and Critical Care Medicine, Third Military Medical University Southwest Hospital, Chongqing, China; ^5^ Department of Pulmonary and Critical Care Medicine, Xi’an International Medical Center Hospital, Xi’an, China; ^6^ Department of Pulmonary and Critical Care Medicine, Shaanxi Provincial People’s Hospital, Xi’an, China

**Keywords:** asthma, airway inflammation, airway remodeling, metformin, AMPK

## Abstract

Metformin has been involved in modulating inflammatory state and inhibiting cell proliferation and angiogenesis. This study aimed to determine whether metformin alleviates airway inflammation and remodeling of experimental allergic asthma and elucidate the underlying mechanism. We sensitized and challenged mice with ovalbumin (OVA) to induce allergic asthma. During the challenge period, metformin was administered by intraperitoneal injection. By histopathological and immunohistochemical analyses, metformin-treated mice showed a significant alleviation in airway inflammation, and in the parameters of airway remodeling including goblet cell hyperplasia, collagen deposition and airway smooth muscle hypertrophy compared to those in the OVA-challenged mice. We also observed elevated levels of multiple cytokines (IL-4, IL-5, IL-13, TNF-α, TGF-*β*1 and MMP-9) in the bronchoalveolar lavage fluid, OVA-specific IgE in the serum and angiogenesis-related factors (VEGF, SDF-1 and CXCR4) in the plasma from asthmatic mice, while metformin reduced all these parameters. Additionally, the activity of 5′-adenosine monophosphate-activated protein kinase *a* (AMPK*α*) in the lungs from OVA-challenged mice was remarkably lower than control ones, while after metformin treatment, the ratio of *p*-AMPK*α* to AMPK*α* was upregulated and new blood vessels in the sub-epithelial area as evidenced by CD31 staining were effectively suppressed. These results indicate that metformin ameliorates airway inflammation and remodeling in an OVA-induced chronic asthmatic model and its protective role could be associated with the restoration of AMPK*α* activity and decreased asthma-related angiogenesis.

## Introduction

Asthma is considered as a heterogeneous disease with two typical features, airway chronic inflammation and remodeling, and affects around 334 million people worldwide (Papi et al., 2018). Asthmatics especially severe ones who are unresponsive to conventional asthma therapy, often suffer high medical costs, impaired quality of life and even early death (Nunes et al., 2017). Fully understanding the precise pathophysiology of asthma is crucial to better asthma control.

Airway inflammation in allergic asthma is mainly characterized by T helper 2 (Th2) cells hyper-activation in response to allergens, followed by infiltration and activation of multiple inflammatory cells with the secretion of inflammatory cytokines (Boonpiyathad et al., 2019). Such a long-lasting inflammatory state could result in structural changes termed remodeling (Guida and Riccio, 2019). The airway remodeling comprises goblet cell hyperplasia, subepithelial fibrosis, collagen deposition, increased airway smooth muscle (ASM) mass and vascularity (Fehrenbach et al., 2017). Transforming growth factor-*β* (TGF-*β*), matrix metalloprotein 9 (MMP-9) and vascular endothelial growth factor (VEGF) are key mediators, promoting these structural changes (Hur and Broide, 2019; Lambrecht et al., 2019). Notably, the pathophysiological changes of both airway inflammation and remodeling process are closely linked with increased vasculature in the sub-epithelial space of airways (Harkness et al., 2015). Neovascularization promotes a persistent influx of inflammatory cells and mediators, as well as provides more oxygen, nutrients and growth factor for abnormal cell proliferation and hyperactivity (Pałgan and Bartuzi, 2015; Eldridge and Wagner, 2019). VEGF and lung-homing of endothelial progenitor cells (EPCs) driven by stromal cell derived factor-1 (SDF-1) binding CXCR4 receptor are crucial to bronchial vascularity in asthma (Imaoka et al., 2011; Janssens et al., 2018; Laddha and Kulkarni, 2019). Hence, novel approaches for asthma treatment should attempt to target both chronic inflammation and remodeling of asthmatic airways.

Metformin, a classical first-line oral hypoglycemic agent, not only plays a beneficial role in metabolic syndrome and cancer, but also shows promise in protecting against chronic inflammation and tissue-remodeling (Bharath and Nikolajczyk, 2021; Wu et al., 2021a). The protective role of metformin on inflammation and remodeling mainly depends on the activation of AMPK, consisting of one *a* catalytic subunit (*α*1 or *α*2) and two regulatory subunits (*β* and *γ* subunits), and primarily regulating cellular energy metabolism (Kim et al., 2016). Emerging evidence favors the notion that AMPK serves as a “metabolic brake” on the energy-consuming processes that drive inflammation and fibrosis, such as diapedesis, chemotaxis, overactive effector T cells and structural cells, and the production of various cytokines (Ursini et al., 2018; Pålsson-McDermott and O'Neill, 2020). Previous *in vivo* and *in vitro* studies have shown that metformin mitigates pulmonary inflammation and fibrosis induced by various injuries, such as lipopolysaccharide (LPS), cigarette smoke and bleomycin (Rangarajan et al., 2018; Wu et al., 2018; Polverino et al., 2021). Besides, metformin alleviates airway inflammation in obese asthma through regulating metabolic parameters and immune response (Guo et al., 2021). However, the action of metformin in allergic asthma and its possible mechanisms are still poorly understood.

Moreover, recent observational studies of patients demonstrated that metformin use, independent of glycemic control and obesity, reduced the risk of asthma exacerbation among patients with asthma and diabetes (Wu et al., 2021b) and improved pulmonary outcomes among individuals with asthma-chronic obstructive pulmonary disease (COPD) overlap but not COPD alone (Wu et al., 2021c). Therefore, we conceived that metformin could alleviate the pathologic manifestation of chronic allergic asthma alone. Using an inhalation model of ovalbumin-induced chronic asthma, we investigated the effects of metformin on asthma-related inflammation and remodeling, and explored its underlying mechanism.

## Materials and Methods

### Ovalbumin-Induced Asthma in Mice

Mice were provided by the Animal Center of Fourth Military Medical University (Xi’an, China) and were bred in specific pathogen-free and temperature-controlled conditions with a 12-h light-dark cycle and allowed free access to standard food and water. First, 6-8-week-old female BALB/c mice were randomly divided into three experimental groups: 1) Control group; 2) OVA group; 3) OVA + MET group. Eight mice were used in each group. To establish a chronic asthma model, mice were intraperitoneally sensitized on day 0 and day 7 with 100 μg ovalbumin (OVA, Grade V, Sigma-Aldrich, St. Louis, MO, United States) emulsified in 1.5 mg aluminum hydroxide (Sigma-Aldrich) in a total volume of 200 μL. From day 21 on, the OVA-sensitized mice were exposed to aerosolized 5% OVA (Grade Ⅱ, Sigma-Aldrich) in a chamber connected to a nebulizer (Omron, NE-C29B, Dalian, China) for consecutive 8 weeks: three times a week, 30 min for each time. In the OVA + MET treated group, the OVA-sensitized mice were injected with metformin (250 mg/kg, Sigma-Aldrich) intraperitoneally 30 min before each inhalation challenge. The control group was subject to the same protocol with saline only.

Twenty-four hours after the last challenge, all mice were euthanized with an overdose of pentobarbital sodium through intraperitoneal injection to collect blood, BAL fluid and lung tissues. All animal experiments were approved by the Institutional Animal Care and Use Committee of Fourth Military Medical University (Xi’an, China) and performed in accordance with the National Institutes of Health Guide for the Care and Use of Laboratory Animals. The study was carried out in compliance with ARRIVE guidelines.

### Histopathology of Lungs

Lungs were removed after the BAL fluid collection. The left upper lungs were fixed in 4% formaldehyde for 18–24 h and then embedded in paraffin. The right lungs were stored in liquid nitrogen for later use Western blotting. The paraffin-embedded lung sections (4 μm thickness) were cut and stained with hematoxylin and eosin, periodic acid-Schiff, and Masson’s trichrome to assess inflammatory cell infiltration, mucus secretion, and subepithelial fibrosis respectively. At least twenty bronchioles with 100–200 μm of internal diameter were randomly collected and evaluated through a light microscope (×200 magnification, Nikon, Tokyo, Japan). All the quantitative analyses in lung tissues were performed by two experimenters in a blinded manner using Image-Pro Plus v6.0 software (Media Cybernetics, Rockville, MD, United States).

Briefly, the inflammation score was based on a five-point scoring system: normal = 0; a few cells = 1; one layer of cell ring = 2; two to four layers of cell ring = 3; >4 layers of cell ring = 4. The thickness of the airway wall and smooth muscle layer was calculated by airway wall thickness or smooth muscle layer thickness (μm) per length of basement membrane (mm) respectively (Li et al., 2019; Ramos-Ramírez et al., 2020). The mucus score was according to a five-point scoring system, based on the percentage of goblet cells in the epithelium: no goblet cells = 0; <25% = 1; 25–50% = 2; 51–75% = 3; >75% = 4. The collagen deposition area stained with Masson’s trichrome was measured by tracing between the outer extent of the collagen layer in the submucosal region and basement membrane. Results are expressed as the area of Masson’s staining per length of basement membrane (μm) (Li et al., 2019).

### Immunohistochemistry of Lungs

Tissue sections were dewaxed and rehydrated in xylene and ethanol solutions. Antigen retrieval of these sections was performed in a 10 mmol/L sodium citrate buffer through microwave and endogenous peroxidase was blocked by 3% hydrogen peroxide. After being blocked by 3% bovine serum albumin or normal rabbit serum for 30 min, the lung sections were incubated with primary antibodies against *a*-SMA (1:2000; Servicebio, Wuhan, China), TGF-β (1:500; Servicebio), MMP-9 (1:1,000; Servicebio), CD31 (1:150, Servicebio) and *p*-AMPK*α* (Thr^172^) (1:100; Cell Signaling Technology, Danvers, MA, United States) at 4°C overnight. All sections were labeled by goat anti-rabbit/mouse or rabbit anti-goat antibody and visualized using a DAB Kit (DAKO, Copenhagen, Denmark). The intensity of the brown color in the peribronchial area was quantified in a blinded method using Image-Pro Plus software (Media Cybernetics, Rockville, MD, United States).

### Immunofluorescence of Lungs

Permeabilization and blocking were done before incubation with goat polyclonal CD31 antibody (1:50; Servicebio, Wuhan, China) at 4°C overnight. The lung sections were then stained with CY3-labeled donkey anti-goat IgG secondary antibody (1:300; Servicebio) for 1 h at room temperature. The tissue slices were washed and mounted with a medium containing DAPI. Fluorescent signals were then recorded by fluorescence microscope and digital photomicrograph (Nikon, Tokyo, Japan) and quantified in a blinded manner through Image-Pro Plus software (Media Cybernetics, Rockville, MD, United States).

### Western Blotting

Frozen lung tissues were weighed (100 mg) and homogenized in 1,000 μl RIPA buffer containing protease inhibitors (CW Biotech, Beijing, China). Lysates were centrifuged at 12,000 rpm for 20 min at 4°C and the protein concentration of the supernatant was quantified by BCA assays (Beyotime, Shanghai, China). Samples were separated by 10% SDS-PAGE and transferred to PVDF membranes (Millipore, Billerica, MA, United States) by the wet transfer method. Membranes were blocked with 5% skim milk solution and incubated with anti-*p*-AMPK*α* (Thr^172^), anti-AMPK*α* at 1:1,000 dilution (Cell Signaling Technology, Danvers, MA, United States) and anti-GAPDH at 1:2,500 dilution (CW Biotech) overnight at 4°C. The corresponding HRP-conjugated anti-rabbit IgG or anti-mouse IgG was incubated at room temperature for 1 h to detect primary antibodies. Blots were visualized with enhanced chemiluminescence reagents (Millipore) and analyzed by ImageJ software (National Institutes of Health, Bethesda, MD, United States).

### Differential Cells and Cytokines in BALF

Immediately after blood collection, BAL was performed with 2 ml phosphate-buffered saline (PBS) through a tracheal cannula and samples were centrifuged at 1,500 rpm for 8 min at 4°C. The cell pellets were resuspended in 200 µl PBS. Total cell counts were enumerated using a hemocytometer and the percentages of differential cells were determined in a cytospin slide stained with Diff-Quick (Sysmex, Kobe, Japan) by counting 200 leukocytes on randomly selected areas of the slide using light microscopy (Olympus, Tokyo, Japan).

The supernatants of BAL fluid were collected to determine the levels of interleukin−4, −5, −13, TNF-α, TGF-*β*1 and MMP-9 by using an ELISA kit (Multisciences Biotech, Hangzhou, China). Briefly, according to the manufacturer’s instruction, the OD value of the solution was obtained at 450 nm by a microplate reader (Bio-tek Instrument Inc., United States). The concentrations of the standard preparation were taken as *X*-axis and their OD value as *Y*-axis to draw a curve and obtain the equation. The OD values of the samples were applied to the equation to calculate the concentration of the samples.

### OVA Specific IgE and Cytokines in Blood

Blood samples were collected by removing eyeball and aliquoted in pyrogen-free tubes for serum and in EDTA anticoagulation tubes for plasma. The levels of OVA-specific IgE in the serum and VEGF, CXCR4, and SDF-1 in the plasma were quantified using a commercial ELISA kit (Westang Biotech, Shanghai, China) according to the manufacturer’s instructions. ELISA plates were read with a Bio-tek microplate reader at 450 nm.

### Statistical Analysis

The data are presented as the mean ± SEM. All statistical analyses were performed using one-way analysis of variance, followed by Turkey’s post hoc test, employing GraphPad Prism v9.0.0 software (GraphPad, San Diego, CA, United States). Differences were considered statistically significant when *p* < 0.05.

## Results

### Metformin Blunts Airway Inflammation in Histology of Lung Tissue in OVA-Induced Mice

**FIGURE 1:**
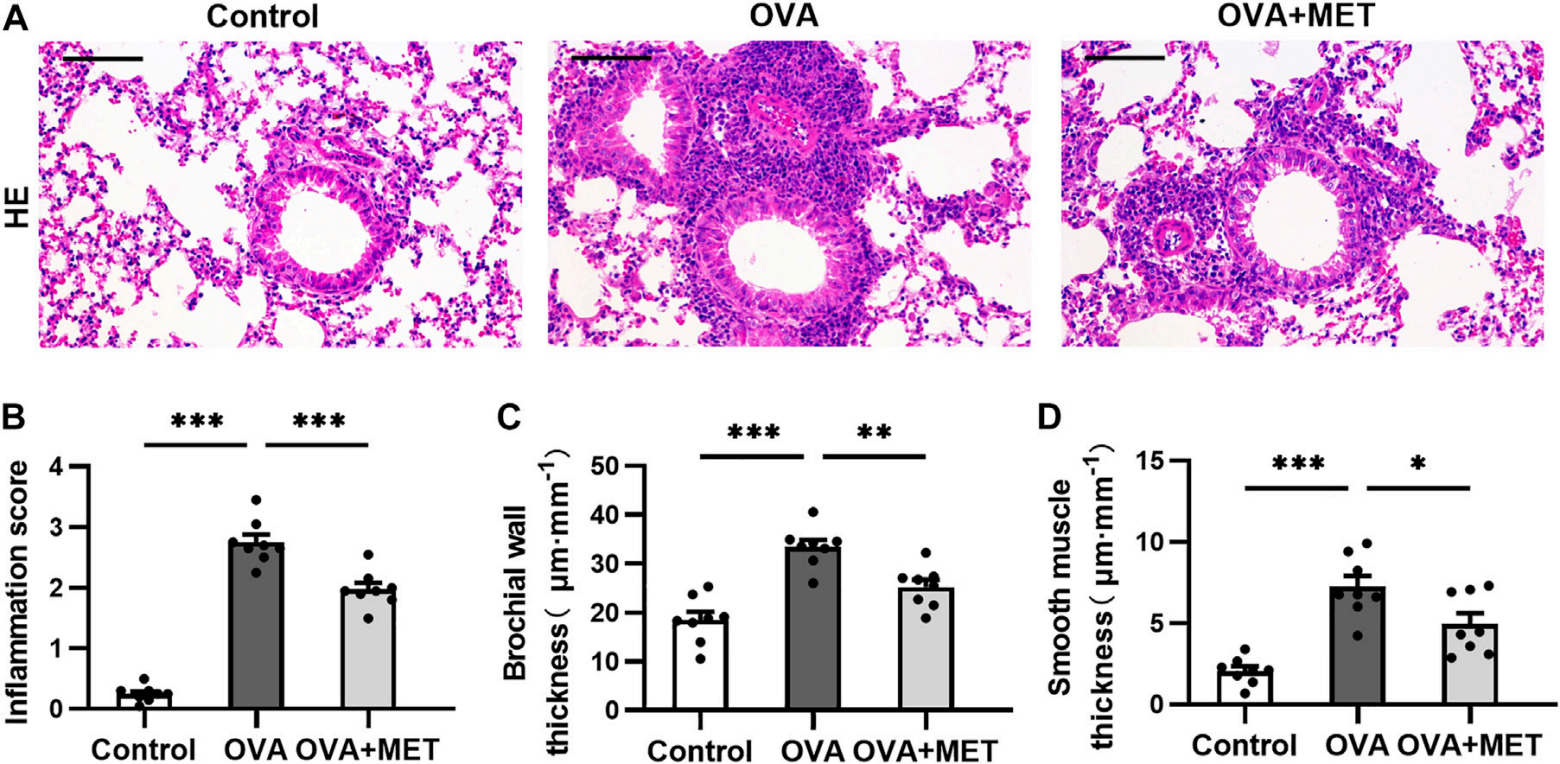
Metformin inhibits inflammatory infiltration in lungs of OVA sensitized and challenged mice. **(A)**. Representative images of HE-stained lung tissue sections from each group. **(B)**. Blinded inflammation score evaluated from HE staining. **(C,D)**. Measurement of airway wall thicknesses and smooth muscle thicknesses from HE-stained lung sections by using Image-Pro Plus software. Data were presented as mean ± SEM (*n* = 8 animals, 20 bronchioles/3 sections of an animal, each bronchiole with 100–200 μm of internal diameter). All images are at ×200 magnification. Scale bar = 100 μm *p < 0.05, **p < 0.01, ***p < 0.001.

Histopathology of hematoxylin-eosin (HE)-stained sections showed increased peribronchial inflammatory cellular infiltration in the lung parenchyma of OVA-induced mice as compared with the saline controls, while the extent of this infiltration decreased remarkably in the metformin-treated mice (Figure 1A). Accordingly, the score of peribronchial inflammatory cells was significantly lower in the OVA + MET group than that of the OVA group (Figure 1B). Additionally, as compared with the control group, repeated ovalbumin exposure increased the thickness of bronchial wall and smooth muscle layer, which decreased markedly with the treatment of metformin (Figures 1C,D). Results suggest that airway inflammation and remodeling were successfully established in our chronic allergic airway disease model and metformin could alleviate these above changes.

### Metformin Attenuates Airway Remodeling in Histology of Lung Tissue in OVA-Induced Mice

To further evaluate the effect of the metformin on airway remodeling in the OVA-sensitized/challenged mice, we conducted histopathological staining with Periodic Acid-Schiff (PAS), Masson’s trichrome and immunohistochemistry (IHC) to evaluate mucus production, collagen deposition, airway smooth muscle (ASM) proliferation and airway fibrosis respectively.

**FIGURE 2:**
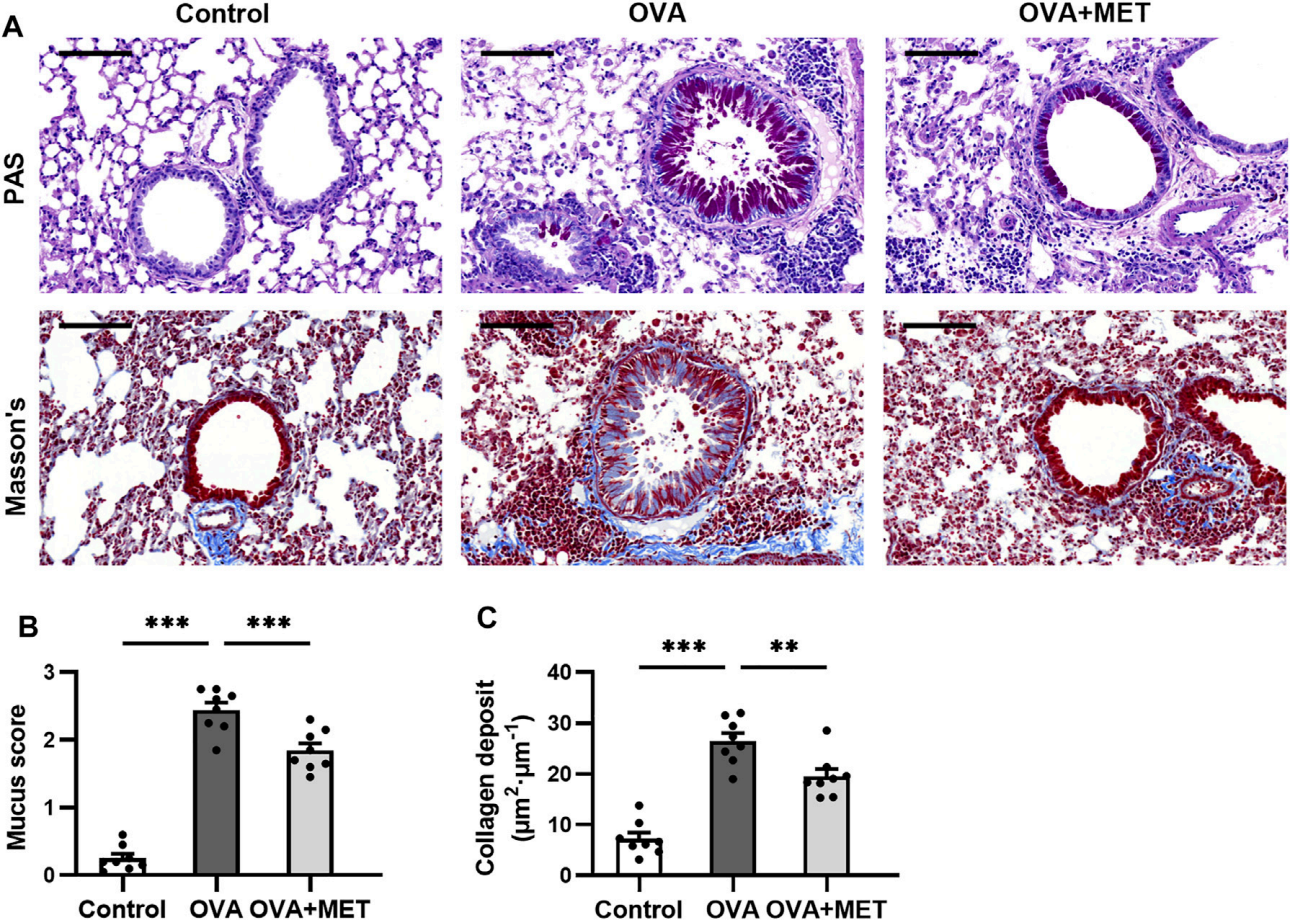
Metformin attenuates airway remodeling in histology of OVA sensitized and challenged mice. **(A)**. Representative images of PAS-stained and Masson’s trichrome-stained lung sections from each group. **(B)**. Blinded scoring of percentage of PAS positive epithelial cells. **(C)**. Blinded quantitative analyses of area of peribronchial Masson’s trichrome staining by Image-Pro Plus. Data were presented as mean ± SEM (*n* = 8 animals, 20 bronchioles/3 sections of an animal, each bronchiole with 100–200 μm of internal diameter). All images are at ×200 magnification. Scale bar = 100 μm *p < 0.05, **p < 0.01, ***p < 0.001.

PAS-stained sections demonstrated remarkable goblet cell hyperplasia in the OVA group. The hyperplasia was attenuated by metformin treatment (Figure 2A). Consistently, the mucus score was significantly higher in the OVA group compared to the OVA + MET group (Figure 2B). Masson’s staining revealed that repeated administration of metformin to mice challenged with ovalbumin contributed to a significant reduction in the area of collagen deposition around the bronchioles (Figures 2A,C).

**FIGURE 3:**
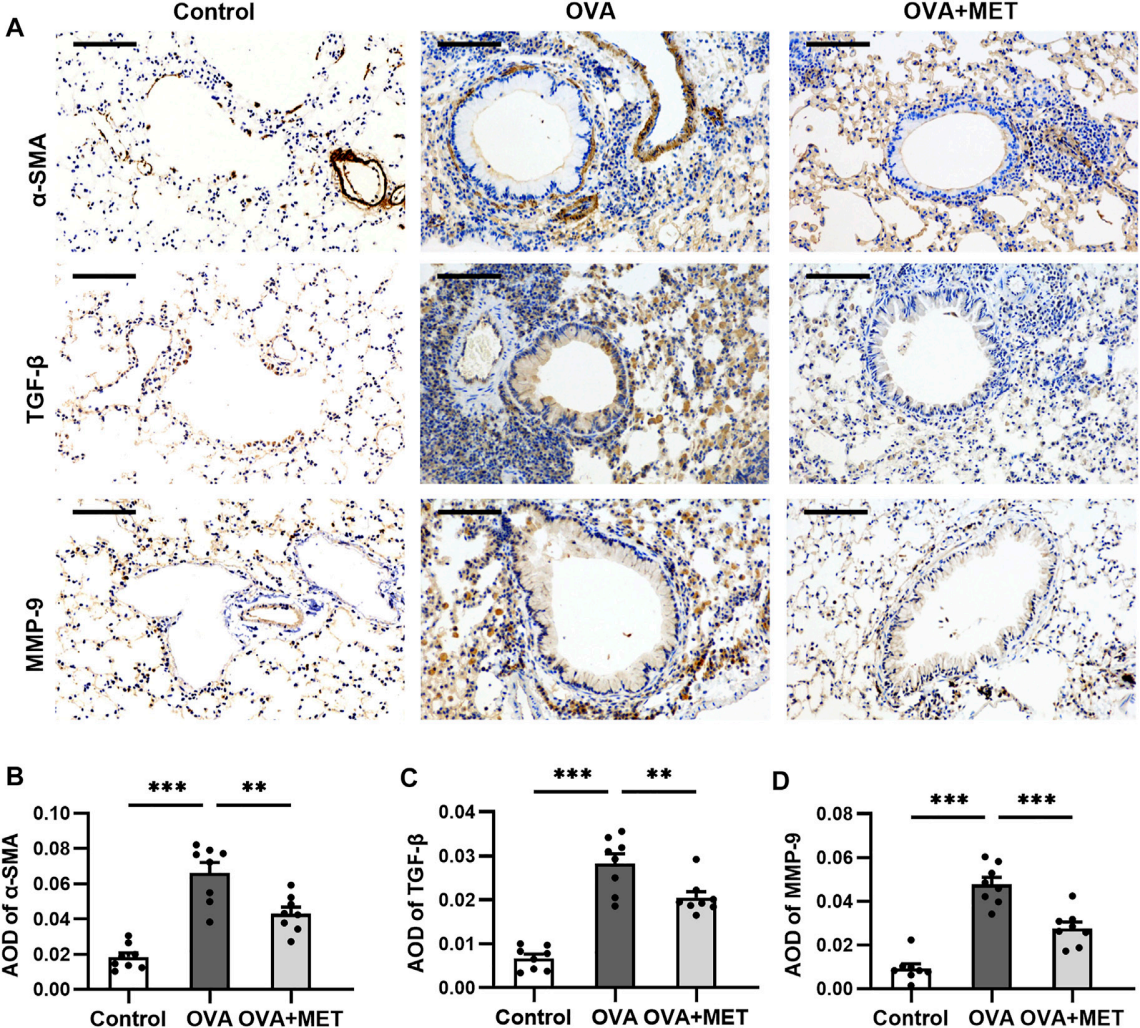
Metformin has an anti-fibrotic effect in a murine model of chronic asthma. **(A)**. Representative images of *a*-SMA, TGF-*β* and MMP-9 immunoreactivity in lung tissues. **(B–D)**. Blinded quantitative analysis of immunohistochemical staining density [(integral optical density)/area; IOD/area] for *a*-SMA, TGF-*β* and MMP-9 by Image-Pro Plus. Data were presented as mean ± SEM (*n* = 8 animals, 20 bronchioles/3 sections of an animal, each bronchiole with 100–200 μm of internal diameter). All images are at ×200 magnification. Scale bar = 100 μm *p < 0.05, **p < 0.01, ***p < 0.001.

Immunostaining of alpha-smooth muscle actin (*α*-SMA) showed that chronic OVA exposure increased the area of *a*-SMA immunostaining compared to the control group. This effect was alleviated by metformin (Figures 3A,B). The expression of immunoreactivity for airway fibrosis-associated proteins TGF-*β* and MMP-9 were significantly elevated in the OVA-induced mice as compared with the saline controls. In the OVA + MET group, the expression of these proteins decreased significantly (Figures 3A,C,D).

These findings indicate that metformin effectively attenuates the main characteristics of airway remodeling in this experimental asthma model.

### Metformin Inhibits the Production of Various Cytokines in the BALF and Plasma, and OVA-Specific IgE in the Serum of Asthmatic Mice

**FIGURE 4:**
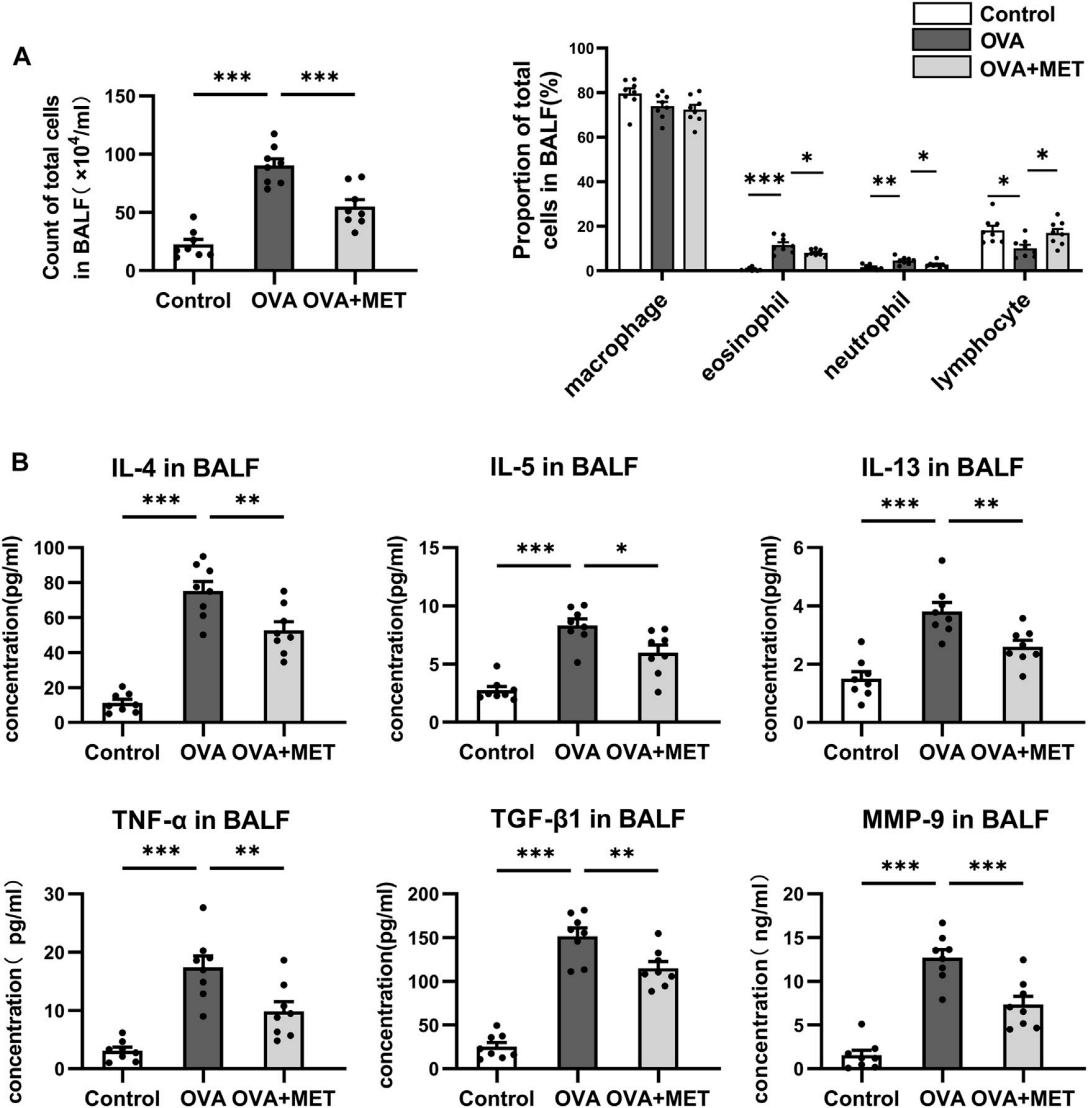
Metformin reduces multiple cytokine levels in the BALF from the asthmatic mice. **(A)**. Comparison of total cell numbers and differential cell counts in the BALF. **(B)**. Comparison of cytokine concentrations (IL-4, IL-5, IL-13, TNF-α, TGF-*β* and MMP-9) in the BALF. Data were presented as mean ± SEM. n = 8. *p < 0.05, **p < 0.01, ***p < 0.001.

To assess the infiltration of cells and cytokines in the lung, bronchoalveolar lavage fluid (BALF) was used. Two months of OVA challenge induced a significant increase in the number of total cells, and percentages of eosinophils and neutrophils. Intervention with metformin significantly reduced the number of total cells, the proportions of neutrophils and eosinophils in the BALF (Figure 4A). Accordingly, the Th2 cytokines (IL-4, IL-5, IL-13), TNF-α, TGF-*β* and MMP-9 in the BALF were also detected. The concentrations of IL-4, IL-5 and IL-13, as well as TNF-α, TGF-*β* and MMP-9 were significantly higher in the OVA-sensitized and challenged mice than those in saline controls, whereas these cytokines reduced markedly in the metformin-treated mice (Figure 4B).

**FIGURE 5:**
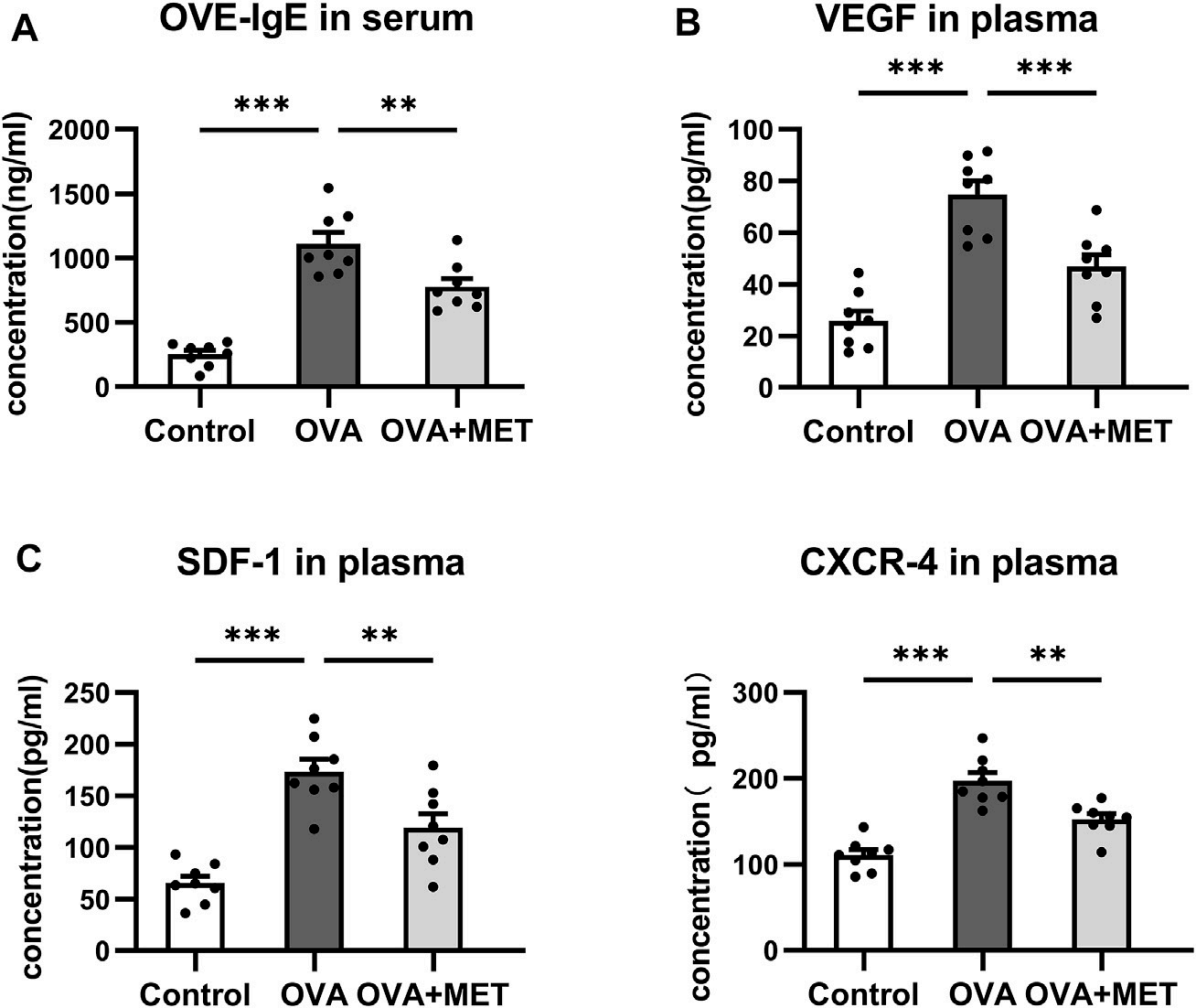
Metformin reduces the levels of OVA-IgE and angiogenesis related cytokines in the blood. **(A)**. Comparison of OVA-IgE concentration in the serum. **(B)**. Comparison of VEGF, CXCR4, and SDF-1 concentrations in the plasma. Data were presented as mean ± SEM. *n* = 8. *p < 0.05, **p < 0.01, ***p < 0.001.

Moreover, ELISA for blood samples revealed that the concentrations of OVA-specific IgE (OVA-IgE) in serum and angiogenesis associated factors including VEGF, stromal cell derived factor-1 (SDF-1) and C-X-C motif chemokine receptor (CXCR4) in plasma were significantly increased in the asthmatic mice as compared with the control ones. These changes were significantly attenuated in the OVA + MET group (Figures 5A–D).

Results indicate that metformin has an inhibitory effect on the Th2 response, as well as the production of many pro-inflammatory, pro-fibrosis and pro-angiogenic factors.

### Metformin Restores AMPK*α* Activity and Impairs Asthma-Related Angiogenesis in the Lungs of Asthmatic Mice

**FIGURE 6:**
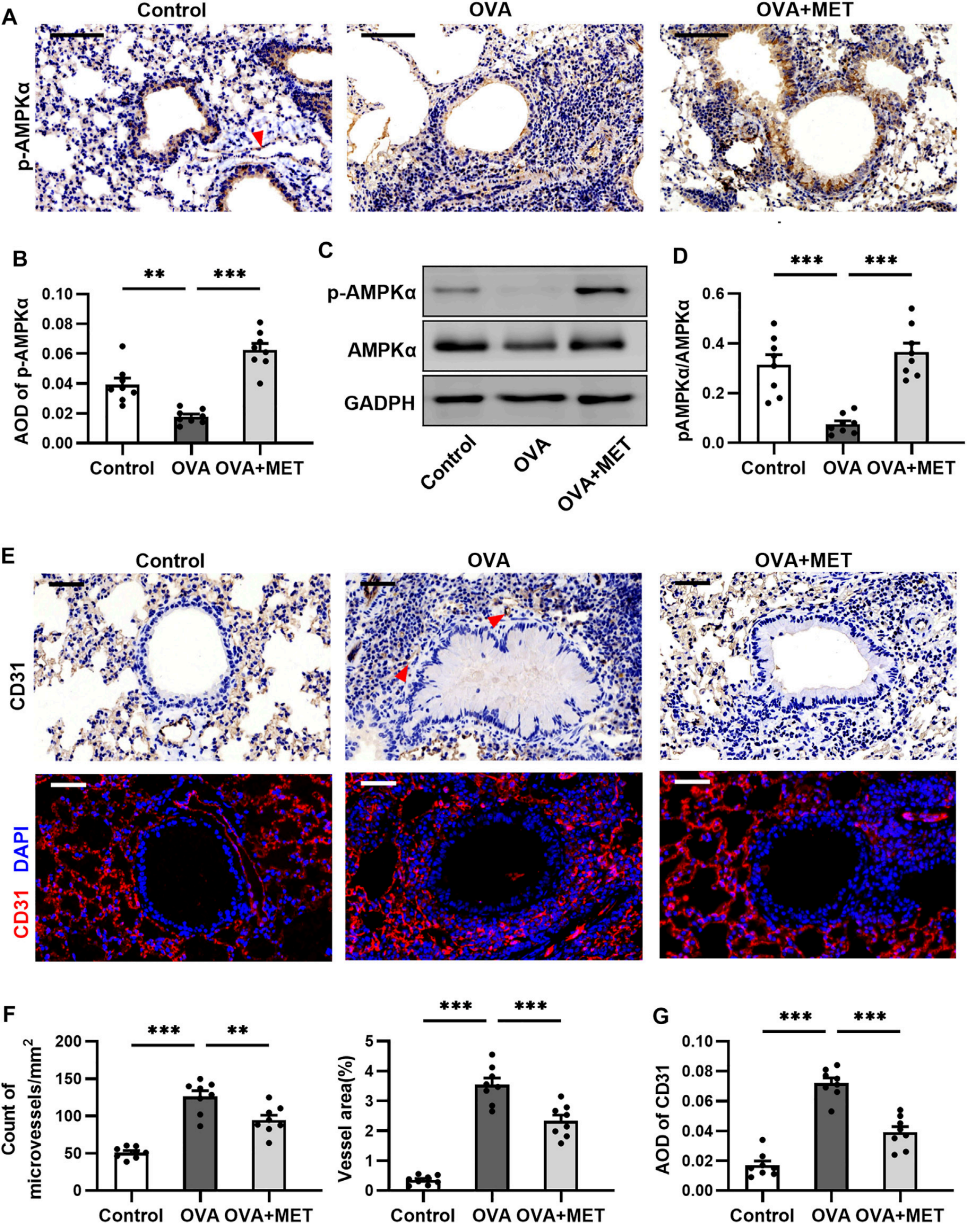
Metformin restores AMPK activity and impairs angiogenesis in the sub-epithelium in lungs of asthmatic mice. **(A,B)**. Representative images and blinded quantitative analyses of *p*-AMPK immunoreactivity in lung sections from each group. The red arrow points to the vascular endothelium stained with p-AMPK*α*. **(C,D)**. Representative Western blots and densitometric analyses of *p*-AMPK and AMPK in lung tissues from each group. **(E)**. Representative images of microvessels with anti-CD31 immunohistochemical and immunofluorescent staining of lung sections. Red arrows point to microvessels stained with CD31. **(F)**. Blinded quantitative analysis of the number and area of microvessels positive for CD31 immunostaining in the sub-epithelial region. **(G)**. Blinded quantitative analysis of fluorescence intensities for CD31 staining in the sub-epithelial region. Data were presented as msean ±SEM (*n* = 8 animals, 20 bronchioles/3 sections of an animal, each bronchiole with 100–200 μm of internal diameter). All images are at ×400 magnification. Scale bar = 50 μm *p < 0.05, **p < 0.01, ***p < 0.001.

To further explore the potential mechanisms of action of metformin, the expression levels of AMPK*α* and *p*-AMPK*α* (phosphorylated at Thr^172^ of its *a* subunit) were determined by immunohistochemical staining and Western blotting in the lung tissues. In the lung sections obtained from control and asthmatic mice, *p*-AMPK*α* staining was found in epithelial cells and endothelial cells, with remarkably fewer *p*-AMPK*α*-positive cells in the tissues from asthmatic mice. After metformin intervention, a prominent increase was observed (Figures 6A,B). Consistently, Western blotting demonstrated that the expression of *p*-AMPK*α* decreased markedly in the OVA group as compared with control group, but significantly increased in the OVA + MET group. Simultaneously, the total level of AMPKα did not vary evidently among these three groups (Figures 6C,D). We thus infer that metformin could facilitate the phosphorylation level of AMPK*α* in epithelial cells and endothelial cells of lung tissues in our experimental asthma mice model.

Furthermore, we wished to ascertain if AMPK*α* activation is involved in angiogenesis in the sub-epithelium of asthmatic mice. Immunohistochemical staining for CD31 showed that the OVA-induced mice exhibited significantly increased number and area of microvessels in the sub-epithelium when compared with control ones, while metformin stunted these increases (Figures 6E–G). Similarly, the quantitative analysis of immunofluorescence intensity for CD31 (red) in the airway sub-epithelium demonstrated a significant decrease in the metformin-treated mice as compared with the asthmatic mice (Figures 6E,H). These results show that activating AMPK*α* could inhibit the angiogenesis around the bronchioles.

## Discussion

This is the first study, to our knowledge, to demonstrate that metformin effectively suppresses both the airway inflammation and remodeling process in an OVA-induced murine model of chronic asthma by restoring AMPK*α* activity. Our animal experiment demonstrated that metformin significantly reduced pathologic changes in the lungs due to inflammatory cells recruitment, inflammatory mediator spillover, goblet cell hyperplasia, ASM hypertrophy, subepithelial fibrosis and neovascularization. Moreover, we observed that the mouse airways exposed to long-term OVA were largely deficient in AMPKα activity, which was recovered by metformin. Together, these results will provide experimental evidence for novel therapeutic strategies in asthma.

Initially, our histological result showed a protective role of metformin in pulmonary inflammation of asthmatic mice, in accordance with previous studies (Calixto et al., 2013; Guo et al., 2021; Ma et al., 2021). However, in their studies, they used obese or acute asthma models and studied the short-term effect of metformin alone or in combination. As the airways of asthma are in a state of chronic inflammation and a long period of intervention can better reveal long-term efficacy for asthma, we adopted a chronic allergic asthma mouse model to investigate the action of metformin alone.

In chronic asthma, airway remodeling is the main pathological feature. In our *in vivo* experiment, pulmonary tissue biopsy using PAS, Masson and *a*-SMA straining demonstrated that metformin abated goblet cell hyperplasia, collagen deposition below the sub-epithelial layer and increased ASM mass. Consistent with a previous study, they reported that metformin reduced pivotal characteristics of airway remodeling in response to ovalbumin and fungal-associated allergenic protease (FAP) (Park et al., 2012). Also, several previous studies confirm that metformin suppressed ASM cell proliferation induced by multiple stimuli *in vitro* and inhibited pulmonary fibrosis in experimental lung fibrosis (Liu et al., 2016; Sato et al., 2016; Rangarajan et al., 2018; Pan et al., 2020). Thus, we further validate its anti-fibrotic role in asthma by identifying two critical remodeling mediators, TGF-*β* promoting extracellular matrix (ECM) deposition and MMP-9 degrading ECM. The immunohistochemical and ELISA results show that metformin significantly reduced the local expressions of both TGF-*β* and MMP-9. Seemingly contradictory, but it is plausible that high degradation with an excessive ECM deposition may be involved in the process of pathological sub-epithelial fibrosis in chronic asthma and metformin could prevent this active fibrotic remodeling. Additionally, our immunohistochemistry and immunofluorescence results show that metformin hindered the emergence of new blood vessels in the sub-epithelial region, thus lessening inflammatory exudation and reversing the progressive airway remodeling process. Overall, these data indicate that metformin may be an effective strategy to resolve airway remodeling in asthma.

Th2-driven inflammation is the defining feature of asthma. In a murine model of chronic asthma, we observed that metformin efficiently reduced infiltration of eosinophils and neutrophils, and production of Th2 cytokines and OVA-specific IgE, which partly fall in line with previous studies in obese or acute asthma models (Guo et al., 2021; Ma et al., 2021). We also found a significant reduction of local levels of non-Th2 cytokines including TNF-α, TGF-*β*1 and MMP-9 after metformin treatment. Similar findings were reported in other disease models, in which metformin inhibited the up-regulation of TNF-α and TGF-*β*1 via targeting multiple cytokine pathways (Gamad et al., 2018; Wu et al., 2019). Furthermore, our data showed that metformin suppressed the systematic expressions of VEGF, SDF-1 and CXCR4 in the plasma, thus mitigating neovascularization and inflammatory exudate in asthma. Earlier studies suggest that metformin decreased the expression of VEGF and mobility of EPC in non-asthmatic disease models (Ambasta et al., 2017; El-Ghaiesh et al., 2020). Nevertheless, the underlying mechanism of metformin as a modulator of angiogenic mediators in asthma needs further investigation. Altogether, these results suggest a potent anti-inflammatory action following metformin administration in asthma and the underlying mechanism is much more complex than inhibiting the simple Th2-skewed immune response.

To further elucidate the role of metformin in alleviating various features of chronic asthma, we explored the distribution and activation level of AMPK. The immunohistochemical result displays AMPK*α* activity was primarily observed in the regions of the airway epithelial cells and vascular endothelial cells from the lungs of control mice, while in OVA-induced mice, a significant decrease in AMPK*α* activity was observed. These data are in accordance with the observation that the protein level of *p*-AMPK*α* was markedly diminished in the OVA-challenged asthmatic lungs, despite similar levels of the total amount of AMPK*α*. This finding is partially inconsistent with a previous study showing elevated levels of both *p*-AMPK*α*1 and total AMPK*α*1 in lungs exposed to OVA and FAP (Park et al., 2012). This inconsistency could be due to the different experimental systems and AMPK subunit tested. Although AMPK *α*1 subunit seems to be widely expressed and extensively studied, some studies indicate that AMPK*α*2 also protected lung endothelial and epithelial barrier function, thereby mitigating lung injury in response to LPS or PM_2.5_ (Xing et al., 2013; Wang et al., 2018). In view of these findings, it is conceivable that loss of AMPK*α* activity may contribute to dysfunction of airway epithelium and vascular endothelium, which can be restored by metformin.

A clear limitation of our present study is the use of an *in vivo* (mouse) modeling system, which just allowed us to dissect the protective role of metformin in asthma at the whole animal level. Therefore, a further detailed mechanistic study using *in vitro* models, is clearly needed to determine how metformin regulates dysfunction of inflammatory cells and structural cells in asthma, and its downstream targets. Moreover, animal models may mimic, in part, the changes seen in human disease. Hence, future research using patients' samples and data is also needed.

In conclusion, findings from our study suggest a potential benefit of metformin that targets both airway inflammation and remodeling in experimental allergic asthma through the restoration of AMPKα activity, which provides a potential therapeutic for asthma.

## Data Availability

The raw data supporting the conclusion of this article will be made available by the authors, without undue reservation.
