# Corrigendum: Metformin Ameliorates Inflammation and Airway Remodeling of Experimental Allergic Asthma in Mice by Restoring AMPKα Activity

**DOI:** 10.3389/fphar.2022.900127

**Published:** 2022-04-21

**Authors:** Wenxian Ma, Qiaoyan Jin, Haiqin Guo, Xinpeng Han, Lingbin Xu, Shemin Lu, Changgui Wu

**Affiliations:** ^1^ Department of Pulmonary and Critical Care Medicine, Xijing Hospital, Fourth Military Medical University, Xi’an, China; ^2^ Department of Biochemistry and Molecular Biology, School of Basic Medical Sciences, Xi’an Jiaotong University Health Science Center, Xi’an, China; ^3^ Department of Pediatrics, The Second Affiliated Hospital of Xi’an Jiaotong University, Xi’an, China; ^4^ Department of Pulmonary and Critical Care Medicine, Third Military Medical University Southwest Hospital, Chongqing, China; ^5^ Department of Pulmonary and Critical Care Medicine, Xi’an International Medical Center Hospital, Xi’an, China; ^6^ Department of Pulmonary and Critical Care Medicine, Shaanxi Provincial People’s Hospital, Xi’an, China

**Keywords:** asthma, airway inflammation, airway remodeling, metformin, AMPK

In the published article, there was an error regarding the affiliation for “Changgui Wu”. Instead of having affiliation “1 and 5”, they should have only affiliation “5”.

The authors apologize for this error and state that this does not change the scientific conclusions of the article in any way. The original article has been updated.

